# Telomeres, Aging and Exercise: Guilty by Association?

**DOI:** 10.3390/ijms18122573

**Published:** 2017-11-29

**Authors:** Warrick Chilton, Brendan O’Brien, Fadi Charchar

**Affiliations:** 1 Faculty of Health Sciences and Faculty of Science and Technology, Federation University Australia, Ballarat, VIC 3350, Australia; b.obrien@federation.edu.au; 2 Department of Physiology, University of Melbourne, Melbourne, VIC 3010, Australia; 3 Department of Cardiovascular Sciences, University of Leicester, Leicester LE1 7RH, UK

**Keywords:** telomeres, aging, physical activity, biomarker, association

## Abstract

Telomeres are repetitive tandem DNA sequences that cap chromosomal ends protecting genomic DNA from enzymatic degradation. Telomeres progressively shorten with cellular replication and are therefore assumed to correlate with biological and chronological age. An expanding body of evidence suggests (i) a predictable inverse association between telomere length, aging and age-related diseases and (ii) a positive association between physical activity and telomere length. Both hypotheses have garnered tremendous research attention and broad consensus; however, the evidence for each proposition is inconsistent and equivocal at best. Telomere length does not meet the basic criteria for an aging biomarker and at least 50% of key studies fail to find associations with physical activity. In this review, we address the evidence in support and refutation of the putative associations between telomere length, aging and physical activity. We finish with a brief review of plausible mechanisms and potential future research directions.

## 1. Introduction

The global population of persons aged 80 years or older will triple by 2050 [1], leading inexorably to a public health imperative. Current increases in life expectancy exceed concomitant increases in disease free years, creating a compression of chronic disease burden in old age [2]. Physiological aging is characterized by cumulative deleterious changes to biological function [3] and is the strongest non-modifiable predictor of most chronic diseases. Despite this, significant variability exists in the health impact of aging [4]. 

The associations between mortality and traditional biomarkers such as blood pressure, cholesterol and body mass index (BMI) weaken with age [5]. The search for a definitive aging biomarker is encumbered by the heterogeneity of cellular aging. Post-mitotic cells are not subjected to the replicative stresses experienced by mitotic cells; therefore, some tissues exhibit greater biological aging than others. The highly variable human lifespan highlights that the mere passage of chronological time is not an effective, isolated measure of aging. Biological aging refers to processes that proceed independently of chronological aging that reduce organismal viability and increase vulnerability. Telomeres are regarded by many as the heir apparent of aging biomarkers, recording both chronological and biological age [6,7]. 

## 2. Telomere Biology—A Primer

Telomeres are specialized DNA structures that bookend nuclear DNA protecting it from degradation [8]. Mitotic division imposes a progressive loss of telomeric base pairs (bps) due to the inability of DNA polymerases to fully replicate the lagging C-strand [9,10]. This cumulative loss simultaneously chronicles replicative history and imposes a finite replicative limit. Critical shortening of telomeres causes eventual disruption of the protective protein shelterin complex [11]. Cells requiring high replicative capacity express telomerase, a specialized ribonucleoprotein complex that synthesizes telomeric repeats [12]. Human telomerase consists minimally of two core components; a reverse transcriptase catalytic subunit (hTERT) and an antisense RNA template (hTERC) [12,13]. Telomeres are sensitive to a host of stimuli including oxidative stress [14], chronic inflammation [15], BMI [16], smoking [17], alcohol intake [18], perceived stress [19] and physical activity (PA) [20]. A growing body of evidence also indicates that telomeres are responsive to habitual PA [20,21]. 

Despite the telomere’s popular designation as a mitotic clock, the relationship between telomere length and aging is inconsistent and does not meet the requisite biomarker criteria [22]. Closer examination of the association with PA also reveals inconsistencies and methodological confounders. The clinical and public interest in the PA-telomere association is predicated upon several tacit assumptions: (i) mean telomere length is causally associated with biological aging and age-related pathologies and (ii) PA can lengthen mean telomere length and that in doing so; (iii) PA will reduce biological aging and disease burden. This review summarizes the current evidence for and against telomeres as aging biomarkers of aging and potential mediators of exercise-induced health gains. 

## 3. Cellular Senescence

Cellular senescence is the progressive and irreversible loss of replicative capacity in somatic cells [23]. Cellular senescence sits astride several paradoxical and seemingly incongruent binary functions; namely tissue regeneration [24] and tissue dysfunction [25], embryonic development [26] and organismal aging [27] and tumor promotion [28] and suppression [29]. Senescence is triggered when the telomeric terminal restriction fragment (TRF) reaches a mean length of 4–7 kb [30]. At this critical threshold, the protective shelterin complex is disrupted exposing an uncapped double-stranded chromosome end. This in turn triggers the ataxia telangiectasia mutated (ATM)-p53-p21 axis and subsequent DNA damage response (DDR) [31], preventing progression into the S-phase of the cell cycle [32,33,34].

Replicative senescence refers to proliferative exhaustive driven by telomere loss whilst stress-induced senescence can be precipitated by oxidative or genotoxic stress, regardless of telomere length [35]. Replicative senescence is causally implicated in aging and age-related diseases such as cardiovascular disease (CVD) [36], diabetes [37,38], osteoarthritis [39], glaucoma [40] and cataracts [41]. In addition to lost proliferative capacity, cellular senescence causes significant changes in gene expression, epigenetic factors and cell morphology [42]. Senescent cells also acquire a characteristic secretome known as the Senescence-Associated Secretory Phenotype (SASP) [43]. This largely pro-inflammatory phenotype can initiate senescence in young cells contributing to tissue dysfunction [25], progression of atherosclerosis [36], cancer [44] and diabetes [37,38].

Replicative exhaustion within the immune system is called immunosenescence; a cluster of age-related changes resulting in decreased replicative capacity [45,46], shortened telomeres [47], increased cytokine production [48,49] and increased susceptibility to infectious diseases [50]. Given the diverse physiological ramifications, senescence may be the causal nexus linking the indirect, microscopic causes of aging with the direct, macroscopic effects of aging [27].

## 4. Telomeres and Aging

A clear inverse association exists between chronological age and telomere length. Support of the causal association between telomeres and aging comes from accelerated aging conditions such as Dyskeratosis Congenita, Werner’s Syndrome and Hutchinson-Gilford Syndrome. Such progeroid syndromes are characterized by a sequela of age-associated pathologies precipitated by accelerated telomere attrition [51,52,53,54,55].

The American Federation of Aging Research stipulate that any candidate aging biomarker must satisfy the following criteria [56,57]:

1. It must predict the rate of aging and therefore be a better predictor of lifespan than chronological age
2. It must reflect and monitor the physiological processes underlying aging
3. It must be a repeatable, unobtrusive and harmless measure
4. It must be testable in animal models

### 4.1. Criterion 1—Must Predict the Rate of Aging Better than Chronological Age

Human telomere length varies by 5000 to 15,000 bps at birth [58], a measure that exceeds total average leukocyte telomere length (LTL) shortening throughout adult life [59]. Genome-wide association studies have identified a host of potential candidate loci associated with LTL variation [60,61,62,63,64,65]. Estimates of telomeric bp loss vary between 30–200 bps per division [66,67]. Although variable in magnitude, age-related decreases in LTL are consistently observed [68]. Three recent studies estimated the annual telomere shortening rate at 17.4 [69], 15.6 [70] and 48–67 bps per chronological year [71]. Paternal age at conception of the offspring is associated with longer offspring LTL [72,73,74], yet infant LTL is positively associated with adult telomere attrition rate [75,76]. However, the annual adult attrition rate contributes less to population LTL variation than LTL variation at birth [77] and attrition rate during the first two decades of life [78,79]. Unlike chronological age, telomere shortening is not linear; the majority of shortening occurs during the rapid somatic expansion from birth to puberty [80,81]. Thereafter, adults with either longer or shorter than average LTL tend to maintain that classification into old age [82,83]. Telomere shortening can be heterogeneous within a given cell as longer telomeres shorten faster than shorter ones [84,85,86]. 

Paradoxically, telomere lengthening has been observed in several longitudinal studies [75,76,87,88,89,90,91,92,93,94,95]. When assessed over a 2–6 year period, most individuals shorten or maintain telomere length; however, 15–44% demonstrate an increase in mean LTL [87,89,96]. The assessment time course appears important as less lengthening is observed when assessed over a 10 year period [97]. Post-intervention increases in telomere length may be due to actual telomerase-mediated addition of telomeric DNA or an apparent lengthening due to redistribution of immune cell subsets [98]; a caveat seldom declared in telomeric research. 

More people are living into old age; however, these people eventually die within a very narrow time span as the finite biological limit of human lifespan is reached [99]. If telomere length reliably scaled with lifespan, individuals with shorter telomeres would theoretically die off before reaching the limits of human lifespan. This would result in an age-associated decrease in telomere length variance known as survivor bias; a phenomenon that has been demonstrated [100,101] and refuted in the data [102]. Most individuals are not living long enough to reach the critically shortened telomere threshold referred to as the telomeric brink. It is believed that further increases in longevity will be limited by telomere length [103]. Given an average starting LTL of 10–15 kilobase pairs, even a yearly loss of approximately 50 bps should still sustain lymphocyte function well beyond 100 years of age. This appears to support the theory that the length of the shortest telomere, as opposed to the average telomere length, triggers cell cycle arrest, genomic instability and senescence [85]. Overall, LTL appears to add predictive power to health measures but is less effective than chronological age [104]. This may be due to high variability in LTL at birth and throughout the lifespan. 

### 4.2. Criterion 2—The Capacity to Reflect Physiological Processes

Telomeres possess clear biological plausibility as a candidate aging biomarker, reflecting oxidative stress, inflammation, replicative history and cellular senescence. Despite that, associations with age-sensitive functional measures are inconsistent. Several studies have failed to find associations between LTL and lung function [105], grip strength [18], blood pressure [106] and several measures of cognitive function [18,101]. However, a more recent investigation identified associations with lung function, grip strength, pulse pressure, reaction time, general mental ability and general health [104]. The Newcastle 85+ study assessed associations between a panel of 74 candidate biomarkers and four health-status measures in a cohort of 852 individuals aged 85 years. Leukocyte telomere length did not fulfil the criterion of significant association with at least two of the health-status measures [107].

Boonekamp et al. propose that organisms consist of redundancy elements that gradually fail and replace each other as they buffer and accumulate damage. Death occurs when the last of the elements are exhausted. Boonekamp et al. further propose that LTL is better viewed as a biomarker of somatic redundancy than biological aging [108]. The somatic redundancy model fits with two established telomere observations: (i) that longer telomeres, possessing more redundancy units, shorten faster than short telomeres [84] and (ii) telomere shortening only becomes detrimental when a critical threshold is passed [109]. The current evidence indicates that LTL likely reflects pre-pubertal somatic growth and post-pubertal cellular senescence and oxidative stress [58].

### 4.3. Criterion 3—A Harmless Repeatable Measure

Most studies measure mean telomere length in circulating leukocytes as physiological proxy for the target tissue. Conclusions from these studies are predicated upon a supposed correlation between LTL and target tissue. One study correlated LTL with vascular tissue telomere length [110] and there is evidence of intra-individual correlation in telomere length between different tissues [111,112]. A more recent study found correlations with only two (intercostal skeletal muscle and liver) out of twelve human tissues assessed [113]. Experimental data suggests that differences in telomere length between tissues are due to tissue-specific attrition rates [114]; however, these rates appears to regress towards a mean value from adulthood to old age [79].

Mean LTL represents the average telomere length across a heterogeneous cell population. A recent study rank-ordered telomeres as longest in B cells, then CD4+ and CD8+CD28+ T cells (similar lengths) and shortest in senescent CD8+CD28- T cells [115]. Telomerase expression is also heterogeneous within the immune system; being highest in B cells, followed by CD4+ T cells, CD8+CD28+ T cells and lowest in CD8+CD28- T cells [115]. Exercise is known to transiently modulate the relative proportions of leukocyte subsets in an intensity-dependent fashion [116]. Therefore, the cellular composition at any given time point will determine the mean LTL and telomerase activity [115].

Considerable variance exists within and between telomere measurement techniques. At present, several different protocols can be used to measure telomere length [117]. These range from the original gold standard Southern blot analysis of terminal restriction fragment (TRF), quantitative polymerase chain reaction-based techniques (qPCR), through to single telomere length analysis (STELA), flow and quantitative fluorescence in situ hybridization (flow FISH and Q-FISH respectively). Each has its own strengths, limitations and coefficient of variation (CV), making comparisons between studies potentially inaccurate. Data from qPCR and Southern blot are weakly correlated (*r* = 0.52) [118]. Comparison between qPCR and flow FISH obtained a similarly weak correlation coefficient (*r* = 0.47) [119]. Next generation sequencing (NGS) technology has given rise to new methods of telomere length measurement. The two key proposed methods both count short reads that contain telomeric repeats [120] and are appropriate for assessment of mean telomere length in genome [121]. However, there is considerable variation between NGS methods and qPCR-based measurements at present [122]. Differences in DNA extraction protocol also significantly influence telomere length [123]. Similarly, telomerase can also be assessed using multiple protocols and variants thereof, either directly measuring telomerase products or signals from telomerase-mediated DNA [124]. 

The likelihood of measurement error in telomere research is high. The inter-individual variation in LTL can be 5000 to 15,000 bps [58] whilst the yearly shortening rates can vary between 30–100 bps [125]. The majority of telomere research utilizes qPCR which has a CV of 6.45% compared to TRF which has a CV of 1.74% [117]. Such variation is likely to render many associations non-significant or questionable at the very least.

### 4.4. Criterion 4—Testable in Animal Models

Animal models have significantly furthered the understanding of telomere homeostasis and disease; however, some inherent limitations affect direct inter-species comparisons. The life expectancy of a mouse is more than 40 times shorter than that of a human yet mouse telomeres are 5 to 10 times longer [126]. The telomerase enzyme is functionally active in the majority but not all, murine tissues [127]; as distinct from humans that lack detectable telomerase levels in many somatic cells [128]. The telomerase-negative mTR^−/−^ mouse was initially developed to examine the role of telomerase in normal and neoplastic growth [129]. It has since made valuable contributions to the understanding of telomere regulation. However, in addition to exhibiting telomere dysfunction and increased end-to-end fusions [129,130], late generation mTR^−/−^ mice can also exhibit a host of other pathological phenotypes [130,131,132,133,134,135,136,137,138,139]. Knock-in of telomerase eliminates many of the degenerative phenotypes observed in late generation mTR^−/−^ mice [140]. The comparatively sterile laboratory conditions mice are subjected to remove several negative telomere instigators such as variable diets, pollution, ultraviolet light and inflammation [141]. 

Whilst rodents are the most widely used animal models, zebrafish have also been used extensively in telomere/telomerase research. Their short life, relatively short generation time and an unlimited capacity to regenerate their fins in 7–10 days makes them a convenient model [142,143]. Expression levels of zebrafish *TERT* mRNA closely correlate with telomerase activity and in accordance with most marine species, they appear to maintain telomerase expression in somatic tissues [144]. A telomerase-mutant zebrafish strain has been widely used to study aging phenotypes; however, it demonstrates aging phenotypes that are far more pronounced than wild-type animals [145]. Telomere/telomerase has also been investigated in avian species [146], primate species [147], plants [148,149], nematodes [150,151] and Drosophila [152]. Each animal model has made contributions to the understanding of telomere homeostasis; however, most have physiological discontinuities that challenge their representativeness of normal human aging.

## 5. Telomeres and Age-Related Diseases

Variable associations exist between accelerated telomere shortening and age-related diseases such as CVD [96,153,154], cancer [155], stroke [156] diabetes [157,158,159], dementia [160,161,162], chronic obstructive pulmonary disease [163] and skin disorders [164]. Shorter LTL was initially associated with coronary artery disease (CAD) in 2001 [165]; with similar CVD associations to follow [106,153,166,167,168,169,170,171,172,173]. Short LTL but not attrition rate, was recently associated with carotid atherosclerosis progression [174]. Moreover, short telomeres were more strongly associated with early-onset than late-onset atherosclerosis. A host of CVD risk factors have also been observationally associated with shortened LTL including smoking [17], diabetes [158], hypercholesterolemia [175], hypertension [176], obesity [177], physical inactivity [20], alcohol consumption [178] and psychological issues [179]. Several other studies have not found associations with blood lipid status [15], hypertension [15,153], smoking [101,180] and BMI [101,180]. A more recent study found no associations between LTL and coronary risk factors including cholesterol, triglyceride, HDL-cholesterol, LDL-cholesterol, smoking, personal or family history of CVD [181].

Three key studies have since failed to find any association between LTL and early atherosclerosis [173,182,183]. Additionally, a recent study associated long, as opposed to short LTL with a nearly three-fold higher risk of developing myocardial infarction [184]. Support for a causal link continues to come from genetic and observational prospective studies using Mendelian randomization to reduce the likelihood of reverse causation. A cluster of seven alleles associated with shortened LTL are themselves associated with a 21% increased CAD risk per standard deviation in LTL [185,186,187]. Despite an abundance of conflicting evidence, the broader scientific consensus is that shortened LTL represents an increased risk of CVD and likely reflects accelerated leukocyte turnover due to oxidative stress and inflammation.

Accumulating evidence indicates an independent pro-telomeric effect of statin treatment [188]. Aspirin decreases oxidative stress and forestalls endothelial cell senescence [189] yet inhibits telomerase activation in polymorphonuclear neutrophils from carotid plaques [190]. Angiotensin-converting enzyme (ACE) inhibitors also demonstrate a pro-telomeric effect via upregulation of *TERT* mRNA in endothelial cells [191]. Androgen treatment also significantly increased LTL in a cohort with telomere-shortening diseases [192].

The relationship between LTL and cancer is complex and at times paradoxical. A detailed treatment of the role of telomeres in malignant transformation is beyond the scope of this review. What follows is a brief overview of current evidence. Telomere shortening and uncapping (loss of shelterin integrity) are believed to play an anticancer role [193]. Long LTL is associated with increased risk of several cancer types [194,195,196,197,198,199,200,201,202,203]. However, in some instances shortened telomeres can potentiate cancer by fusing with other uncapped telomeres, thereby creating genome destabilizing fusion-bridge-breakage cycles [193]. Telomerase activity is a critical component in malignant transformation with 85–90% of all malignant tumors being telomerase positive [128,204]. It is estimated that ~15% of human cancers maintain telomere length through one or more mechanisms referred to as Alternative Lengthening of Telomeres (ALT) [205]. A cluster of seven alleles associated with LTL homeostasis, TERC, TERT, oligonucleotide/oligosaccharide-binding fold containing one gene (OBFC1), zinc finger protein 208 (ZNF208), regulator of telomere elongation helicase 1 (RTEL1), acylphosphatase 2 (ACYP2) and nuclear assembly factor 1 ribonucleoprotein (NAF1) are simultaneously associated with CAD [185] and cancer [195,198]. If the alleles result in comparatively long telomeres, the cancer risk is elevated and the CAD risk is reduced; the reverse is also true. It has been proposed that the cancer protection conferred by short telomeres represents an evolutionary trade-off resulting in decreased proliferative and regenerative capacity [206]. Non-linear U-shaped relationships have been observed between telomere length and several cancer risk profiles [207]. This may in part be explained by the destabilizing and potentially oncogenic effects of shortened telomeres [208] and the increased replicative capacity and potential accumulation of abnormalities associated with longer telomere length [209].

One proposed explanation for the discrepant associations is that telomere uncapping has an anti-cancer effect in the young but a potentially pro-cancer effect in the elderly [210]. Hypothetically, the more robust telomere dysfunction-based mechanism of the young would prevent tumorigenesis whilst the shortened, uncapped and depleted telomeres of the elderly may allow bypass of aberrant cells [210].

## 6. Telomeres, Longevity and Mortality

The association with overall longevity is similarly inconsistent. Whilst several studies support an association with longevity [211,212,213,214], just as many refute it [101,180,215,216]. However, the observation that LTL is longer in women may be linked to the greater longevity observed in women [217]. Additionally, in twin studies, the individual with longer LTL exhibits enhanced longevity compared to the other twin [218].

A range of studies have identified age-adjusted inverse associations between mean LTL and all-cause mortality [69,88,160,162,211,212,219,220,221,222,223,224,225,226,227]. However, other studies were unable to replicate the association in similar cohorts [18,91,96,101,153,180,215,216,228,229,230]. Analysis of cohorts from Costa Rica, Taiwan and United States of America found that after adjustment for gender and age, LTL ranked between 15th and 17th out of 20 established predictors and biomarkers of all-cause mortality [231]. A cohort of 4576 healthy individuals had LTL measured twice within a 10-year interval and were then monitored for morbidity and mortality for another 10 years. Change in LTL and risk of all-cause mortality were not significantly associated [95]. Despite abundant associations between telomere length and age-associated mortality, the association diminishes with age [108]; failing to predict mortality in cohorts of the very old [101,180]. Paradoxically, telomere length in early childhood most accurately predicts life expectancy [108,232].

## 7. Physical Activity and Telomere Length

The widely touted relationship between PA and LTL is replete with inconsistencies. Support for a positive association has come from a range of observational/cross-sectional studies [20,179,223,233,234,235,236,237,238,239,240,241,242,243,244,245,246,247,248]. A summary of human studies that significantly associated PA with telomere length is contained in Table 1. Telomere length has been assessed in skeletal muscle cells and white blood cells (WBCs) in response to aerobic training, resistance training and self-reported PA. The observed associations appear hormetic, with low and excessive levels of activity associated with shorter telomeres [241,244] and moderate levels more commonly associated with longer LTL [223].

A 2016 study of 6474 males and females positively associated running with LTL yet found no associations with other PA domains including aerobics, basketball, bicycling, dancing, running, stair climbing, swimming, walking and weight-lifting [249]. The authors speculate that the sustained weight-bearing status of running may preferentially activate signaling pathways, citing other studies that demonstrated associations in ultra-endurance runners [234]. One can only speculate that insufficient statistical power explains the lack of association found in other studies employing running as the independent variable [250,251]. Self-reported PA was positively associated with LTL in a cohort of 5823 men and women from the National Health and Nutrition Examination Survey (NHANES) [70]. Objectively measured maximal cardiorespiratory fitness (VO_2max_) has also been positively associated with LTL in a cohort of obese women [252] and older exercise-trained participants [239].

The positive associations have been refuted by several observational and interventional studies [15,89,97,250,251,252,253,254,255,256,257,258,259,260,261,262,263,264,265,266]. A summary of human studies that found no significant association between PA and telomere length is contained in Table 2. A rigorous 2015 systematic review and meta-analysis concluded that insufficient quality evidence exists to conclusively associate PA with LTL [267]. Approximately 54% of studies reviewed found no relationship between PA and LTL; 41% found a positive association and 5% identified a curvilinear relationship [267]. Most of the positive associations were weak to moderate with only two studies reporting strong associations [223,234]. The analysis cited methodological issues such as weak correlations, assessment of varied tissue types, arbitrary cuff-off points, inadequate blinding of researchers, selective inclusion of other potentially confounding lifestyle factors and discrepant measurement techniques as possible confounders [267]. Discrepant modes of PA and the wide-spread use of self-reported PA with its inherent biases may also explain the lack of association in several of the studies. A similar review conducted in 2013 assessed the effect of exercise in animals and humans and identified three general association types: positive association, inverted “U” response and no association [21].

Whilst some studies identify beneficial chronic effects of exercise on telomere length, acute telomere shortening has also been observed within the same cohort [268]. A study of 2006 Chinese participants found no significant difference in LTL across quartiles of PA [266]. Crucially, the author cited possible decreased role of PA in the seventh decade given potential selection bias of recruiting healthy elderly participants. A common criticism of studies failing to find associations with PA is the lack of statistical power; however, a range of studies with sample sizes ranging from 1942–5862 participants have failed to find an association between PA and LTL [15,95,253,264,265,266]. In a cohort of 4576 Danish men and women, PA was not associated with LTL change over 10 years [95]. A study by Sun et al. (2012) did not demonstrate an association between PA and LTL in a cohort of 5862 middle-aged women [264]; however, the addition of five low-risk factors (smoking status, PA, adiposity, alcohol use and diet) to the analysis established a significant association.

## 8. Proposed Mechanisms

Despite the positive associations between habitual PA and LTL, a clear mechanistic understanding of telomeric adaptation is lacking. Following is a summary of the more widely held mechanistic theories and some candidate mechanisms still subject to further investigation. For a detailed review of other candidate molecules and signaling pathways, readers should seek out the relevant reviews [21].

## 9. Telomerase and Physical Activity

Longer telomeres are often inferred by increased telomerase activity; however, levels do not reliably correlate with telomere length [276]. In a study of 124 health individuals, telomerase activity progressively decreased in concert with telomere length from ages 4 to 39 years. However, 65% of individuals aged 40 years or older exhibited low yet stable telomerase expression despite continued telomere shortening [277]. 

Mouse models have demonstrated exercise-induced telomerase increases [247,278,279,280]; however, human results are conflicting. In one study, there was no difference in peripheral blood mononuclear cell (PBMC) telomerase expression between physical fitness categories [241]. Yet in another study, endurance-trained athletes demonstrated increased leukocyte telomerase compared to controls [247]. A 3 month multifaceted intervention consisting of diet, exercise and stress management techniques increased PBMC telomerase expression; however, direct causation cannot be assigned to one specific modality [281]. Whilst some interventional studies using acute and intensive exercise interventions failed to increase leukocyte telomerase expression [260], a recent study elicited increased PBMC telomerase expression after a single bout of treadmill running [282]. Exercise-induced increases in *hTERT* mRNA have been observed; however, a correlation with telomerase enzyme activity can only be inferred [283].

Telomerase preferentially targets short telomeres [284], therefore acute increases in telomerase may be an attempt to stabilize critically shortened telomeres [285]. This may explain the increased telomerase activity and shortened telomeres that accompany subclinical coronary atherosclerosis [286]. 

## 10. Oxidative Stress

The accumulation of oxidative stress-induced DNA damage is a key factor in cellular dysfunction and organismal aging [287], neurodegeneration [288,289], atherosclerosis, diabetes [290] and carcinogenesis [291]. Telomeric DNA is particularly susceptible to oxidative damage due to the telomeric G triplet [14,292,293,294]. Chronic oxidative stress decreases telomeric DNA repair mechanisms [295], resulting in cumulative and irreparable damage [35]. In doing so, telomeres chronicle chronic exposure to oxidative stress [296]. 

Paradoxically, mild exposure to oxidative stress serves a telomere protective function by increasing anti-oxidant defense systems [262,297,298,299,300,301] and enhancing oxidative damage repair systems [302]. Cellular antioxidant capacity is correlated with cellular proliferative capacity and telomere attrition rate [303]. Overexpression of superoxide dismutase reduces telomere shortening in human fibroblasts [304]. Additionally, under conditions of oxidative stress, telomerase translocates to the mitochondria where it appears to play a protective function [305,306]. The association between habitual moderate intensity exercise and longer telomeres [223,273] may be mediated by an exercise-induced increase in antioxidant defenses. However, a study of obese middle-aged women demonstrated increases in antioxidant enzymes without concomitant changes in LTL [262].

## 11. Inflammation

The PA-mediated attenuation of chronic inflammation likely underpins the protective effect of PA on LTL. Chronic inflammation causes hematopoietic stem cell activation and subsequent WBC turnover resulting in telomere attrition [307]. Shortened LTL have been associated with elevated concentrations of pro-inflammatory cytokines interleukin (IL)-6 and tumor necrosis factor (TNF)-α [308]. Chronic inflammation can also elicit replicative senescence, oxidative stress and modulation of telomerase activity [309,310,311,312,313]. Shorter mean LTL may reflect an increased burden of senescent cells which are known to create a pro-inflammatory phenotype [314,315]. Despite the association, chronic inflammation levels but not LTL predicted successful aging in a cohort of 1554 centenarian, supercentenarians and very old individuals [228].

A consistent inverse association is seen between habitual PA and chronic inflammation [316,317]. The inverted “U” response of telomere homeostasis identified in some studies may be partially explained by the hormetic inflammatory and oxidative stress response to exercise [318,319]. Whether PA decreases inflammation and subsequent telomere attrition or decreases telomere attrition, therefore forestalling the onset of senescence-mediated inflammation, remains an open question.

## 12. The Shelterin Complex

Mouse models have demonstrated exercise-induced plasticity of shelterin components including *Trf2* mRNA [279] and skeletal muscle *Trf1* mRNA [320]. Human studies, mostly observational, have identified increased *TRF2* mRNA and protein in young and middle-aged athletes compared to controls [247]. Increased mRNA expression of leukocyte adrenocortical dysplasia homolog (*TPP1*) and *hTERT* mRNA was also observed in athletes compared to controls [254]. Athletes who performed seven marathons in one week demonstrated upregulated DNA damage repair enzymes Ku70 and Ku80 in addition to increased *TRF1*, *TRF2* and *Pot-1* mRNA expression. This occurred without concomitant changes in telomere length or telomerase activity [260]. A single 30 min bout of treadmill running upregulated WBC *hTERT* and telomeric repeat binding factor 2 (*TERF2IP*) mRNA expression in healthy males [283]. Whilst highly plausible mechanisms, these observations do not infer causation.

## 13. Epigenetics and Telomere Homeostasis

Telomere length is influenced by a host of epigenetic factors including methylation of sub telomeric DNA [321,322,323], histone modification [324,325], posttranslational modifications of shelterin components [326,327] and non-coding RNAs [328]. An in-depth review of all proposed epigenetic pathways is beyond the scope of this review. What follows is a brief overview of the current evidence.

MicroRNAs (miRNAs) play critical roles in modulating gene expression via translational repression or total degradation of the target mRNA [329]. MiRNAs mediate several exercise adaptations [330,331,332,333,334] and demonstrate exercise-induced expression profiles in leukocyte subsets [335,336,337,338,339]. Exercise-induced increases in WBC miR-186 and miR-96 expression were observed after a 30-min exercise bout [283]. In silico analysis identified *TERF2IP* as a potential regulatory target of miR-186 and miR-96. Telomeric repeat factor 1 is translationally repressed by miR-155 resulting in chromosome alterations and telomere fragility [328]. Animal models indicate that miR-155 expression decreases in response to exercise [340]. Expression of *TERT* mRNA is regulated by miR-498 [341]; however, little is known about the exercise responsiveness of miR-498. 

A clear understanding of the role of miRNA-mediated telomere homeostasis is still in its infancy. The direction of the miRNA-telomere interaction is currently unclear; telomere shortening may actually cause differential miRNA expression [342]. Most of the established miRNA-telomere associations have been observed in cancer cell lines, wherein dysregulated telomere maintenance mechanisms may have corrupted the observed associations. To gain a causal understanding of the role of miRNAs in telomere homeostasis, gain/loss of function experiments must be conducted in cell lines and animal models.

Accumulating evidence indicates that global DNA methylation may regulate telomere length variability in adults [343,344]. Associations between DNA methylation and telomere length were initially established in mice [345]; subsequent human studies identified a positive association between global DNA methylation at proximal sub telomeric regions and telomerase activity [346]. Little is currently known about exercise-induced methylation changes within telomeres.

Human telomeres have long been considered transcriptionally silent; however, recent evidence indicates they are transcribed by RNA polymerase II, producing a class of long noncoding RNA (lncRNA) containing telomeric repeats called TERRA [347,348]. TERRA molecules are displaced from telomeres and recruited to chromosomal ends through interaction with the telomeric structure, including shelterin components TRF1 and TRF2 [349]. TERRA also interacts with telomeres via the formation of RNA:DNA hybrid structures known as R-loops [350,351,352,353], which are involved with gene expression regulation [354] and transcription termination [355]. In shortened or damaged telomeres, TERRA is observed in increased levels [356,357,358]. Upregulation of TERRA is also observed in instances of DNA damage or loss of TRF2 [358]. With so much still to learn about lncRNAs such as TERRA, a full understanding of their potential telomere-regulatory role is several years away.

## 14. Conclusions

The proposed associations with aging and exercise are biologically plausible. Telomere length holds tantalizing promise as a biomarker; however, a host of evidential inconsistencies and paradoxes must be addressed. Leukocyte telomere length is highly variable at birth, a metric influenced by genetic inheritance and paternal age at conception. It reflects lifelong exposure to oxidative and inflammatory burden yet childhood LTL has more predictive fidelity than LTL in adulthood or old age. Oscillating throughout the lifespan, even inexplicably lengthening despite advancing age, mean LTL differs between genders. Attrition rates also differ between genders and appear dependent upon initial telomere length. Shortening trajectories can be further influenced by variable exposure to a wide range of environmental stimuli. The association between PA is more questionable with 50% of studies failing to find a significant association. 

Investigations into plausible mechanisms have returned promising yet inconsistent findings. The prevailing consensus is that exercise-induced reductions in oxidative stress and inflammation likely mediate the effect. The possibility that LTL is a physiological epiphenomenon cannot be excluded. Changes in LTL may simply be coincidental processes that reflect, without directly influencing, the primary mechanism. It is likely that telomere length per se is significant only in so much as it reflects the resultant phenotype via pathways such as senescence-associated inflammation. 

It has been proposed that evolutionary pressures have fine-tuned telomere length to reduce the risk of short and long telomere pathologies, namely atherosclerosis and cancer [359,360,361]. The long-term consequences of manipulating telomere length are not well understood and should therefore be approached with equal measures of enthusiasm and evidence. 

## 15. Future Directions

Telomere and telomerase research is on the brink of truly significant contributions to human health and disease management. Exciting associations and plausible putative mechanisms seem to promise therapeutic targets for the future. The extensive associations strongly imply causal involvement and warrant closer, more detailed analysis. We propose four general areas of future research direction.

First, evidence of actual telomere lengthening is lacking. Whether an individual can move from the lowest to the highest quartile of LTL through lifestyle interventions, presumably with an enhanced phenotype, remains an open and essential question. Given the propensity for LTL to track through the lifespan [82], the capacity of PA to modulate a seemingly centrally-driven measure must be addressed. If actual telomere lengthening is identified, it must then be determined whether such lengthening lies within the considerable natural ebb and flow of telomere length dynamics over time. 

The second future direction regards selection of the most relevant telomeric metric. Evidence suggests that the proportion of short telomeres may be more consequential than mean LTL given the ability of very long telomeres within a cell to skew the mean [362,363]. Studies reporting longer LTL in the physically active should also report the relative proportion of the shortest telomeres.

The third future direction regards the use of mediation analysis to quantify positive and negative telomere instigators. Mediation analysis models a hypothesized causal chain in which a variable of interest influences an intervening mediator variable which in turn mediates the outcome variable. This allows the effect of exposure to the mediator on the outcome variable. Mediation analysis quantifies the exposure of likely mediating factors such as oxidative stress, inflammation and age. Analysis of this sort would also reveal whether changes in telomere length can occur independently of factors like oxidative stress and inflammation. As an example, a recent study found that fasting insulin partially mediates the association between genetically determined LTL and CHD risk [364].

The final proposed direction is the development of a regularly updated international consensus document on telomere research methodology. Developed by an international consortium of leading telomere researchers, this document could outline: (i) preferred measurement techniques; (ii) acceptable coefficients of variation; (iii) universally accepted definitions and criteria for long and short telomeres and (iv) rigorous methods of data interpretation. In time, publishing in accordance with such guidelines would become the standard expectation with reputational costs incurred for discrepant methodologies. 

Many exciting discoveries are undoubtedly yet to come from the field; however, many of these may be lost to unacceptably large measurement variations and inconsistent methodologies. The irresistible logicality of the reported telomeric associations risks the premature inference of causation. The final quantum leap to be made will be the translation of benchtop findings into clinically relevant interventions. Only then will the ends have justified the means.

## Figures and Tables

**Table 1:** A summary of studies showing positive associations between physical activity and telomere length.

Ref.	Subjects (*n*)	Tissue	Measurement	Key Findings
[19]	63 healthy post-menopausal women; sedentary group, active group	Leukocytes	T/S qPCR	Sedentary: one unit increase in the Perceived Stress Scale = 15-fold increase in odds of having short telomeres (*p* < 0.05). Active: Perceived stress unrelated to telomere length (*p* = 0.45).
[20]	White twins 2401: 2152 females, 249 males	Leukocytes	Southern blot TRF	Leisure time PA positively associated with LTL (*p* < 0.001). LTLs of the most active subjects were 200 nucleotides longer than least active (*p* = 0.006).
[70]	5823 adult participants; males (*n* = 2766), females (*n* = 3057)	Leukocytes	T/S qPCR	Relative PA (*p* < 0.0002) and absolute PA (*p* = 0.0052) associated with longer LTL after adjustment for demographic variables. Prevalence of short telomeres associated with relative PA (*p* < 0.0001).
[179]	1552 Caucasian female twins: 749 dizygotic twins, 27 monozygotic twins. Distributed into six socioeconomic status (SES) groups	Leukocytes	Southern blot TRF	PA positively associated with TRFL (*p* < 0.005). Overall decreasing trend in TRFL with lower SES (*p* < 0.024). Significant difference in TRFL between non-manual and manual workers (*p* < 0.01).
[223]	44 healthy, post-menopausal women, divided into habitual exercise and sedentary groups	Leukocytes	T/S qPCR	LTL significantly higher in habitual exercise group compared to sedentary group (*p* < 0.01).
[233]	274 pairs same sex twins (153 dizygotic pairs, 121 monozygotic pairs)	Leukocytes	Southern blot TRF	LTL is positively associated with self-reported physical ability in all pairs combined (*p* = 0.006). Positive association between PA and LTL in all pairs (*p* = 0.034).
[234]	67 male ultra-marathon runners, 63 age and sex-matched controls	Leukocytes	T/S qPCR	LTL 11% longer in ultra-marathon runners compared to controls (*p* < 0.001).
[235]	Nurse’s health study—7813 females	Leukocytes	T/S qPCR	Moderately or highly active women had 0.07 SD increase in LTL compared to least active (*p* = 0.02). Greater moderate or vigorous activity associated with longer LTL (*p* = 0.02).
[236]	392 post-menopausal women with Stage I–III breast cancer	PBMCs	Southern blot TRF	No PA significantly associated with shorter LTL (*p* = 0.03).
[237]	895 participants: 476 females, 419 males	Leukocytes	T/S qPCR	Low frequency PA an independent predictor of short LTL (*p* < 0.001).
[238]	944 participants with stable CHD, distributed into three exercise capacity groups: low (*n* = 299), moderate: *n* = 334, high: *n* = 381	Leukocytes	T/S qPCR	LTL significantly longer in subjects with high exercise capacity compared to low (*p* < 0.001). Association remained after adjustment for CVD severity and physical inactivity (*p* = 0.005)
[239]	57 participants stratified into four groups: young sedentary (*n* = 15), young exercising (*n* = 10), older sedentary (*n* = 15), older exercising (*n* = 17)	Leukocytes	Southern blot TRF	LTL of older exercising subjects significantly longer than age-matched sedentary controls (*p* < 0.001). LTL of older exercisers not significantly different from young exercisers (*p* = 0.12). LTL positively associated with VO_2max_ (*p* < 0.01).
[240]	1764 adults: 51% males, 49% females, 73% non-Hispanic whites; distributed into cardiorespiratory fitness tertiles	Leukocytes	T/S qPCR	LTL longer in upper tertile (*p* = 0.04) and middle tertile (*p* = 0.02) compared to lowest tertile.
[241]	69 healthy participants: 34 males, 35 females; distributed into four exercise energy expenditure quartiles	PBMCs	T/S qPCR	Significantly longer telomeres in second exercise energy expenditure quartile compared to first (*p* = 0.001) and fourth (*p* = 0.04) quartiles.
[242]	20 male participants: 5 young athletes, 5 young non-athletes, 5 older athletes, 5 older non-athletes	Skeletal muscle	T/S qPCR	Longer telomeres in older athletes compared to older non-athletes (*p* = 0.04). Young athletes not different to young non-athletes (*p* = 0.12). Strong correlation between VO_2max_ and T/S ratio in athletes (*p* = 0.02).
[243]	239 post-menopausal women	Leukocytes	T/S qPCR	One SD below mean PA levels, major life stressors were associated with LTL shortening (*p* = 0.008). One SD above mean PA level, major life stressors were not associated with LTL shortening (*p* = 0.48).
[244]	782 males: three PA groups low (*n* = 148), moderate (*n* = 398), high (*n* = 236)	Leukocytes	Southern blot TRF	Inverted “U” response. Moderate PA positively associated with longest LTL (*p* = 0.03). LTL the same in low and high PA groups. Moderate PA group had lowest proportion of short LTL (*p* = 0.02).
[245]	46 participants distributed into three PA groups: never trained (*n* = 15), moderately trained (*n* = 16), intensively trained (*n* = 15)	PBMCs	Flow-FISH	T cell TL longer in moderately trained and intensively trained compared to never trained (*p* < 0.05). Significantly longer telomeres in CD8+ T cells in IT group (*p* < 0.05).
[246]	36 healthy participants distributed into three groups: young (*n* = 12), old mobile (*n* = 12), old immobile (*n* = 12)	Skeletal muscle	T/S qPCR	Mean TL from leg muscle of the old immobile group was significantly shorter than old mobile group (*p* < 0.05), young group (*p* < 0.05), arm muscle (*p* < 0.05).
[247]	Young sedentary controls (*n* = 26), young athletes (*n* = 32), middle-aged sedentary (*n* = 21), middle-aged athletes (*n* = 25)	Leukocytes	FlowFISH and T/S qPCR	Older sedentary controls had shorter mononuclear cell telomeres than all other groups (*p* < 0.001). Age-dependent telomere loss attenuated in lymphocytes (*p* < 0.001) and granulocytes (*p* < 0.001) of older athletes.
[248]	667 healthy adolescents: 169 white males, 179 white females, 155 black males, 164 black females	Leukocytes	T/S qPCR	Vigorous PA positively associated with telomere length (*p* = 0.009)
[249]	6474 participants: 49.6% males, 50.4% females	Leukocytes	T/S qPCR	LTL positively associated with running (*p* = 0.03)
[268]	20 endurance athletes, 42 age- and gender-matched controls	Buccal cells	T/S qPCR	Baseline TL better preserved in endurance athletes (*p* = 0.003). Intermediate TL reduced in endurance athletes compared to baseline (*p* = 0.002). Final time point TL reduced in endurance athletes compared to baseline (*p* = 0.0006).
[269]	477 healthy males and females	Leukocytes	T/S qPCR	Vigorous PA positively associated with LTL (*p* < 0.01).
[270]	814 participants: 397 males, 417 females	Leukocytes	T/S qPCR	LTL longer in participants: currently exercising compared to inactive (*p* = 0.013), engaged in intensive activity (*p* = 0.011), participating in sport for 10 years prior to assessment (*p* = 0.017).
[271]	1476 older aged white and African American women	Leukocyte	Southern blot TRF	Highest self-reported PA associated with longer LTL compared to lowest PA (*p* = 0.02). Higher levels of moderate-to-vigorous PA (*p* = 0.04) and faster walking speed (*p* = 0.03) associated with longer LTL.
[272]	582 older adults	Leukocytes	Southern blot TRF	Greater walking distance (*p* = 0.007) and better chair test performance (*p* = 0.04) associated with longer LTL cross-sectionally. Change in chair test performance associated with less LTL shortening longitudinally (*p* = 0.04).

CHD: coronary heart disease. CVD: cardiovascular disease. FlowFISH: fluorescence in situ hybridization combined with flow cytometry. TL: telomere length. LTL: leukocyte telomere length. PA: physical activity. PBMCs: Peripheral blood mononuclear cells. TRF: terminal restriction fragment analysis. TRFL: terminal restriction fragment length. T/S qPCR: the ratio of telomere PCR value to single-copy gene value derived from quantitative PCR. SD: standard deviation. SES: socio-economic status. VO_2max_: maximal volume of oxygen uptake.

**Table 2:** A summary of studies showing no association between physical activity and telomere length.

Ref.	Subjects (*n*)	Tissue	Measurement	Key Findings
[15]	2509 participants: males (*n* = 1218), females (*n* = 1291)	Leukocytes	Southern blot TRF	Self-reported PA not associated with LTL (*p* = 0.806).
[89]	608 individuals with stable CAD: 244 males, 364 females	Leukocytes	T/S qPCR	PA did not independently modulate LTL (*p* = 0.59).
[95]	4576 male and female Danish participants	Leukocytes	T/S qPCR	Change in LTL over 10 years not associated with PA (*p* = 0.85)
[273]	612 advanced prostate cancer cases 1049 age-matched controls	Leukocytes	T/S qPCR	No significant association between PA and LTL in all subjects combined (*p* = 0.262). LTL positively associated with healthy lifestyle factors (e.g., diet, PA, smoking) (*p* = 0.004).
[250]	17 marathon runners 15 age- and sex-matched sedentary controls	Lymphocytes and granulocytes	T/S qPCR	No significant difference between marathon runner lymphocyte TL (*p* = 0.6) and granulocyte (*p* = 0.9) compared to controls.
[251]	18 experienced runners, 19 sedentary individuals	Skeletal muscle	Southern blot TRF	No significant difference in minimum TRF between runners and sedentary (*p* = 0.805). Minimum TRF within runners inversely related to years of running (*p* = 0.007) and hours spent training (*p* = 0.035).
[252]	439 overweight and obese women Randomized into: dietary weight loss (*n* = 118), aerobic exercise (*n* = 117), diet and exercise (*n* = 117), control (*n* = 87)	Leukocytes	T/S qPCR	No significant difference in LTL in exercise group (*p* = 0.51), diet and exercise group (*p* = 0.14). Baseline LTL positively associated with VO_2max_ (*p* = 0.03).
[253]	2284 women (Nurses’ Health Study)	Leukocyte	T/S qPCR	No association between LTL and PA (*p* = 0.69).
[255]	981 individuals: 467 males, 514 females	Leukocytes	T/S qPCR	Work related PA not associated with LTL (*p* = 0.933)
[256]	521 obese individuals randomized into: 2 Mediterranean diet groups, 1 low-fat control group	Leukocytes	T/S qPCR	Self-reported PA not associated with telomere length (*p* = 0.186).
[257]	190 subjects with impaired glucose tolerance, 188 controls	Leukocytes	T/S qPCR	No significant difference in LTL following multi-faceted lifestyle intervention at 4.5 years follow up (*p* = 0.76).
[258]	14 individuals, 7 powerlifters, 7 healthy controls	Skeletal muscle	Southern blot TRF	TL not significantly associated with 8 ± 3 years of powerlifting experience (*p* = 0.07).
[259]	599 healthy males: 392 former athletes, 207 controls	Leukocytes	T/S qPCR	No association between self-reported volume of leisure time PA and LTL in later life (*p* = 0.845).
[261]	42 participants: 10 young males, 6 young females, 13 old males, 13 old females	Skeletal muscle	Southern blot TRF	Tibialis anterior telomere length not significantly influenced by self-reported PA (*p* value not available).
[262]	16 obese middle-aged women randomized into: exercise group, control group	Leukocytes	T/S qPCR	No significant change in LTL after 6 months of aerobic training.
[263]	136 participants: 50 males, 86 females	Lymphocytes	T/S qPCR	Self-reported PA not associated with longer TL (*p* = 0.4570).
[264]	5862 women	Leukocytes	T/S qPCR	No association between PA and LTL. Presence of five low-risk healthy lifestyle factors associated with longer LTL (*p* = 0.015).
[265]	1942 male and female participants	Leukocytes	T/S qPCR	PA not significantly associated with LTL in males or females (*p*-value not available)
[266]	2006 Chinese participants: male (*n* = 976), female (*n* = 1030)	Leukocytes	T/S qPCR	No significant difference in TL across quartiles of self-reported PA (*p* = 0.32)
[274]	84 Caucasian individuals distributed into two groups: exercise trained (*n* = 44), recreationally active controls (*n* = 40)	PBMCs	T/S qPCR	No significant difference in PBMC telomere length between exercising group and controls (*p* = 0.72).
[275]	203 healthy participants; African (*n* = 96), Caucasian (*n* = 107)	Leukocytes	T/S qPCR	Habitual PA not associated with LTL.

CAD: coronary artery disease. LTL: leukocyte telomere length. PA: physical activity. PBMCs: peripheral blood mononuclear cells. TRF: terminal restriction fragment analysis. T/S qPCR: the ratio of telomere PCR value to single-copy gene value derived from quantitative PCR. VO_2max_: maximal volume of oxygen uptake.
